# Could university training and a proactive attitude of coworkers be associated with influenza vaccination compliance? A multicentre survey among Italian medical residents

**DOI:** 10.1186/s12909-016-0558-8

**Published:** 2016-01-29

**Authors:** Claudio Costantino, Emanuele Amodio, Giuseppe Calamusa, Francesco Vitale, Walter Mazzucco

**Affiliations:** Department of Science for Health Promotion and Mother to Child Care G. D’Alessandro, University of Palermo, Via del Vespro n 133, ZIP code 90127 Palermo, Italy

**Keywords:** Influenza vaccination, Medical residents, Multicentre survey, Coworkers attitude, University training

## Abstract

**Background:**

Although influenza vaccination has been demonstrated to be safe and effective, vaccination coverage rates among health care workers and among medical residents appear generally low. Several investigations have been performed worldwide to analyze the healthcare workers’ educational deficiencies.

This multicentre survey aimed to investigate at a nationwide level training quality and work environment associated with seasonal influenza vaccination uptake among Italian medical residents.

**Methods:**

A retrospective cohort study was carried out from April 2012 to June 2012 on medical residents regularly attending the post-graduate medical schools of 18 Italian Universities via an anonymous, self administered, web-based questionnaire. Data have been analyzed by using the R statistical software package.

**Results:**

A total of 2506 out of 10,854 medical residents (23.1 %) have been recruited. The quality of training on influenza and influenza vaccination was reported as “fair” or “poor” during both pre-graduate (40.7 % of respondents) and post-graduate medical school (59.6 % of respondents).

Vaccination uptake was associated with adherence to seasonal 2011/2012 influenza vaccination of medical school tutors (adjusted OR = 4.4; 95 % CI = 1.35–14.26) and other medical residents (adjusted OR = 2.2; 95 % CI = 1.14–4.23).

Moreover, influenza vaccination uptake was also associated with correct knowledge about the virus composition of 2011/2012 influenza vaccine (adjusted OR = 2.43; 95 % CI = 1.64–2.58) and consultation of scientific sources or Institutional recommendations on influenza vaccination (adjusted OR = 6.96; 95 % CI = 3.38–214.36).

**Conclusions:**

Medical residency represents an opportunity to implement educational and training interventions aiming to promote appropriate professional behaviors and skills. Our study suggest that appropriate training, adequate education and proactive coworkers feelings can improve influenza vaccination attitudes towards young doctor.

## Background

Influenza vaccination is universally recognized as the essential intervention to limit the spread of the virus particularly among elderly and patients with comorbidities [1, 2].

Moreover, during annual influenza epidemics, vaccination of the health care workers can contribute to reduce both the spread of the virus to defenceless patients and the absence from work [3, 4].

Unfortunately, despite influenza vaccine has been demonstrated to be safe and effective in healthy persons under 65 years of age, providing 70 % to 90 % protection against infection, influenza vaccination uptake rates observed in different studies among health care workers appear generally low, far away from the recommended rate of 75 % [5–8].

Recently, some studies have examined the influenza vaccination coverage among medical residents and general practitioner trainee physicians in the Italian setting, documenting very low coverage rates (<30 %), in line with other studies conducted worldwide [9–14].

Although health care workers generally recognize the fundamental role of promoting influenza vaccination, in the last years there is an intense debate on the effectiveness of mandatory vaccination among health care workers, especially for its ethical and legal implications [15–17].

Nevertheless, several investigations have been performed at a local level to analyze the healthcare workers’ educational deficiencies and the correlations with the actions taken by public health authorities to implement vaccination rates, however no study has systemized and analyzed such results at a nationwide level [18, 19].

According to the previous considerations, the Italian Medical Residents Influenza Vaccination working group aimed to investigate, via a multicentre web-based survey, training quality, work environment and knowledge associated with seasonal influenza vaccination uptake among Italian medical residents.

## Methods

This retrospective cohort study was carried out on medical residents regularly attending the post-graduate schools of 18 Italian Universities (Bari, Bologna, Brescia, Catania, Catanzaro, Chieti, L’Aquila, Messina, Modena, Napoli Federico II, Palermo, Pavia, Parma, Roma Cattolica, Roma Tor Vergata, Siena, Torino, Verona).

Email addresses of each medical resident were requested and obtained from the Universities information Centers. Medical residents without at least one valid email address were excluded from the study.

Each medical resident included in the study was recruited from April 2012 to June 2012 by receiving an e-mail containing an explanation of the study objectives, an informed consent form and a link to an anonymous, self administered, web-based questionnaire.

The study was approved by the Institutional Review Board of the AOUP “P. Giaccone” Palermo, Italy.

### Questionnaire

The questionnaire was designed by the Italian Medical Residents Influenza Vaccination working group. The first version of the questionnaire was validated in a pilot study carried out on 100 medical residents attending post-graduate medical schools at the University of Palermo. The final version of the questionnaire encompassed 10 sections including 23 items. Data concerning vaccination coverage, reasons of influenza vaccination uptake and refusing, as well as attitudes to recommend influenza vaccination to patients, were analyzed elsewhere [20].

In the present study, we have focused on data related to university and post-graduate training and work environment as reported below:

1. Demographic characteristics: sex, age, year of graduation, year of residency, specialty duties (categorized according to Italian legislation as “Clinical duties” including Allergy and immunology, Cardiology, Dermatology, Endocrinology, Gastroenterology, Geriatrics, Infectious disease, Nephrology, Neurology, Oncology, Pediatrics, Physiatry, Psychiatry, Pulmonology, Rheumatology; “Surgical duties” including Cardiovascular surgery, General surgery, Gynecology, Maxillofacial surgery, Neurosurgery, Ophthalmology, Otolaryngology, Orthopedic surgery, Pediatric surgery, Plastic surgery, Surgical oncology, Thoracic surgery, Urology, Vascular surgery; “Diagnostic duties” including Hygiene and Preventive Medicine, Anesthesiology, Clinical laboratory sciences, Microbiology, Emergency medicine, Forensic Medicine, Intensive care medicine, Pathology, Radiology, Occupational Medicine), geographic setting (categorized as “North”, “Centre” and “South” of Italy);
2. Adherence to 2011/2012 influenza vaccination among coworkers (post graduate medical school tutors and other medical residents): categorized as “Yes, ≤ 90 % of tutors/ medical residents”, “Yes, ≤ 50 % of tutors/ medical residents”, “Yes, ≥ 25 % of tutors/ medical residents”, “None of tutors/ medical residents”, “I don’t know”
3. Training quality on influenza and its vaccination during degree and post graduate medical school: investigated by closed ended question, categorized as “excellent”, “good”, “satisfactory”, “fair”, “poor”;
4. Knowledge about the influenza vaccine virus assortment for 2011-2012 season: investigated by closed ended questions categorized as “AH1N1, AH3N2 and B” (correct answer), “AH1N1, AH5N1, B”, “AH1N1 and H3N2” and “AH3N2 and B” (wrong answers), “I don’t know”;
5. Main informative sources on influenza/influenza vaccination: categorized as “none”, “not interested in any information sources”, “recommendation of National Health Minister”, “recommendation of International Health Authorities”, “scientific sources” and “mass media”, “internet (facebook, twitter, blog, youtube)”, “word of mouth among health care workers”, “other”.

### Statistical analysis

Absolute and relative frequencies have been calculated for qualitative variables, while quantitative variables were summarized as median (interquartile range). Categorical variables were analyzed using the chi-square test (Mantel–Haenszel) and medians were compared by using the Mann-Whitney-Wilcoxon test. All variables were found to have a statistically significant association (two-tailed p-value <0.05) with vaccine uptake in the univariate analysis have been included in a backward stepwise logistic-regression model. Goodness of fit was calculated for each model, and the model with the lowest Akaike Information Criterion (AIC) was considered to have the best fit. Adjusted OR (adj-OR) with 95 % confidence intervals (95 % CIs) have also been calculated for the variables retained in the final model. The significance level fixed for all of the analysis was 0.05, two-tailed. Data have been analyzed by using the R statistical software package [21].

## Results

### Main findings

A total of 2506 out of 10,854 medical residents (24.1 % of the sample) have been recruited in the survey. The median age of medical residents involved in the study was 29 (interquartile range = 3). The majority of the respondents were females (*n* = 1621; 64.7 %). Specialty fields more represented were clinical (1099; 43.9 %), followed by diagnostic (*N* = 840; 33.5 %) and surgical (*n* = 567; 22.6 %). Influenza vaccination rate during 2011/2012 season was 11.9 % (data not shown in table) [20].

As reported in Table 1, the training on influenza and influenza vaccination during the postgraduate medical school education was reported as “fair” or “poor” by 1021 study participants (40.7 %). Similarly, during the postgraduate medical education the training on influenza and its vaccination was reported as “fair” or “poor” by 59.6 % of medical residents (*n* = 777 and *n* = 716, respectively).

**Table 1:** Training quality, coworkers attitudes and knowledge towards influenza and influenza vaccination among Italian medical residents

Explored item or dimension	*n* = 2,506	
	*n*	(%)
Training quality on influenza and influenza vaccination during degree		
- excellent	42	(1.7)
- good	441	(17.6)
- satisfactory	1,002	(40.0)
- fair	784	(31.3)
- poor	237	(9.4)
Training quality on influenza and influenza vaccination during post graduate medical school		
- excellent	49	(2.0)
- good	303	(12.0)
- satisfactory	661	(26.4)
- fair	777	(31.0)
- poor	716	(28.6)
Adherence to influenza vaccination among post graduate medical school tutors		
- ≤ 90 % of tutors	133	(5.3)
- ≤ 50 % of tutors	70	(2.8)
- ≥ 25 % of tutors	509	(20.3)
- none of tutors	144	(5.7)
- do not know	1,650	(65.8)
Adherence to influenza vaccination among other post graduate medical school MRs		
- ≤ 90 % of MRs	53	(2.1)
- ≤ 50 % of MRs	50	(2.0)
- ≥ 25 % of MRs	665	(26.5)
- none of tutors	424	(16.9)
- do not know	1,314	(52.4)
Knowledge about the influenza vaccine virus composition for 2011–2012 season		
- correct information	593	(23.7)
- erroneous information	538	(21.5)
- do not know	1,375	(54.8)
Main informative sources on influenza vaccination		
- none	1,684	(67.2)
- not interested	509	(20.4)
- recommendation of Health Minister/International Authorities	79	(3.1)
- scientific sources	109	(4.3)
- others (mass media, blog, internet, facebook, word of mouth among HCWs, etc…)	125	(5.0)

1658 medical residents (65.8 %) reported to be unaware if their medical tutors during postgraduate medical school were vaccinated against seasonal influenza. At the same time, 1314 medical residents (52.4 %) did not know if their residency colleagues had been vaccinated during influenza season.

Only 23.7 % of the respondent medical residents (*n* = 593) had knowledge about the correct composition of 2011/2012 seasonal influenza vaccine, whereas 54.8 % (*n* = 1375) did not have this information and 21.5 % (*n* = 538) gave a wrong answer.

The majority of survey sample (*n* = 1684; 67.2 %) did not consider any informative sources on influenza vaccination and, in addition, 509 of them (20.4 %) were not interested in any sources of information.

Factors involved in the decision to get vaccinated during the 2011-2012 influenza season have been analyzed in the multivariable analysis reported in Table 2.

**Table 2:** Factors involved in the decision to get vaccinated during 2011–2012 influenza season among Italian MRs (*n* = 2506)

Explored item or dimension	Vaccine uptake during the 2011-2012 season^e^
	Adj OR
	(95 % CIs)
Main specialty duties	
- clinical	Referent
- surgical	0.92 (0.69–1.27)^d^
- diagnostic	1.22 (0.85–1.56)^d^
Adherence to 2011/2012 seasonal influenza vaccination among tutors	
- none of tutors	Referent
- do not know	4.27 (1.32–13.75)^c^
- yes (≤90 %, ≤ 50 %, ≥ 25 %)	4.4 (1.35–14.26)^c^
Adherence to 2011/2012 seasonal influenza vaccination among other MRs	
- none of tutors	Referent
- do not know	1.59 (0.83–3.06)^d^
- yes (≤90 %, ≤ 50 %, ≥ 25 %)	2.2 (1.14–4.23) ^c^
Knowledge about the influenza vaccine virus composition for 2011-2012 season	
- do not know	Referent
- erroneous information	1.46 (0.97–2.21)^d^
- correct information	2.43 (1.64–2.58)^a^
Main informative sources on influenza vaccination	
- not interested	Referent
- none	2.48 (1.38–4.45)^b^
- others (mass media, blog, internet, facebook, etc…)	1.81 (0.77–4.3)^d^
- recommendation of Health Minister/International Authorities, scientific sources	6.96 (3.38–14.36)^a^

^*a*^ *p* < 0.001, ^*b*^ *p* < 0.01, ^*c*^ *p* < 0.05, ^*d*^ *p* ≥ 0.05

^e^Adjusted for age, sex, year of graduation, year of residency, geographic setting, training quality on influenza and influenza vaccination during degree and post graduate medical school

Specialty duty was not significantly associated with influenza vaccination attitude (adjOR for surgical duties = 0.92; 95 % CI = 0.69–1.27, adjOR for diagnostic duties = 1.22; 95 % CI = 0.85–1.56).

Adherence to seasonal 2011/2012 influenza vaccination by postgraduate medical school tutors (adjOR = 4.4; 95 % CI = 1.35–14.26) and other post graduate medical school medical residents (adjOR = 2.2; 95 % CI = 1.14–4.23) was significantly associated with decision to get vaccinated.

Moreover, influenza vaccination uptake was also related to correct knowledge about the virus composition of 2011/2012 influenza vaccine (adjOR = 2.43; 95 % CI = 1.64–2.58).

Lastly, medical residents adhering to vaccination during 2011–2012 season reported to be more prone to consult scientific sources or Institutional recommendations (Health Minister, International Health Authorities) on influenza vaccination (adjOR = 6.96; 95 % CI = 3.38–14.36).

## Discussion

### Interpretation

A proper medical education, both during degree and postgraduate medical school, is vital for the acquisition of adequate professional attitudes and knowledge on preventive choices, based on evidence-based prevention principles [22]. Medical residency represents an opportunity to implement educational and training interventions, aimed at promoting appropriate professional behaviors and modifying inappropriate ones. Unfortunately, our study shows that in Italy, the vaccination coverage against seasonal influenza among medical residents is far below the recommended rates, exactly as observed among other health care workers. Given the documented low influenza vaccination coverage, medical residents represent potential influenza carriers to the patients, especially particularly debilitated subjects and those at risk of hospital-related complications (immunocompromised patients, the elderly, children, pregnant women, etc.) [23].

Thus, medical residents could be considered as a core target group specific educational interventions.

The efficacy of educational and training interventions for improving medical residents’ attitudes towards influenza vaccination has already been proven in different geographic settings [24].

However, our results highlight some other determinants that could play a key role in promoting influenza vaccination compliance. The first of these determinants is specific knowledge on the topic; in Italy, this appears to be as inadequate as the influenza vaccination coverage [25].

In fact, more than 30 % of the medical residents who took part in our study stated that they had received unsatisfactory training on influenza and vaccination against it, both during medical school and postgraduate courses. Our results suggest that the inadequate training during medical residency reported by 30 % of respondents could be a contributing factor responsible for the finding that three out of four respondents either did not know or erroneously identified the vaccine virus composition. Moreover, just over 7 % of the medical residents referred to scientific sources or Institutional recommendations (Health Ministry, American and European Centers for Disease and Control), while indifference prevailed.

Intriguingly, both being aware of the actual composition of the influenza vaccine and consulting scientific informative sources were significantly associated with a greater compliance with influenza vaccination uptake, also after adjustment.

These findings seem to suggest that accurate information about influenza vaccine should be considered as an independent factor associated with higher vaccination coverage among young physicians. Furthermore, our study documents an insufficient education regarding the importance of the vaccine during both undergraduate and postgraduate studies.

All of the previous evidence could also explain why more than half of respondents felt that they should attend multidisciplinary ad hoc courses to improve their knowledge on influenza and vaccinating against it, as reported in a previous paper [20].

Most hospitals/health systems in the US and overseas are implementing a mandatory influenza vaccination protocol and have moved away from voluntary vaccination programs for health care workers, so these results can no longer be generalized. Nevertheless, our data demonstrate that neither mandatory vaccination nor incentives for vaccination are considered as realistic options by the Italian medical residents to implement vaccination adherence, confirming possible ethical and legal consequences and noticeable application difficulties within certain contexts [20, 26–28].

However, having specific knowledge on the topic is not the only factor positively associated with influenza vaccination compliance; an important role is also played by the general work environment. As shown by the multivariable analysis, a proactive coworker attitude, especially involving the tutors (but also among residency colleagues), could represent a key aspect associated with an increased vaccine coverage among health care workers [29].

### Limitations

Major limitations affecting the present study are essentially three. Firstly, the respondents included in the study were only about a quarter of the targeted cohort, so the results are not representative of the majority. Moreover, the low response rate (24.1 %) could be indicative of a selection bias, since respondents with positive personal attitude to influenza vaccination may have predominantly responded to the survey.

Finally, although all the data used in our study were collected anonymously, a potential residual social desirability bias cannot be excluded. However, the consistency between results and vaccination rates documented in the different Italian Regions and different specialty duties lead us to conclude that these possible limitations only marginally affect the results of the study. Furthermore, the influenza vaccination uptake rate is coherent with previous studies conducted among health care workers [6, 18, 30].

Despite of the potential limitations discussed above, this is the first study carried out in Europe that analyzes, on a national level, the impact of a proper training and a favorable work environment on influenza vaccination uptake among young physicians in training.

## Conclusions

According to the recommendations of the American National Vaccine Advisory Committee, stating that “Health Care Employers and facilities should establish comprehensive influenza infection prevention programs that include education of Health Care Personnel as a key component”, our study confirms the need of appropriate training and education in order to improve physicians’ attitudes towards influenza vaccination [31].

The analysis of the collected data suggests some strategies that could be implemented in order to increase influenza vaccination coverage among medical residents. A valid approach could be organizing educational activities on the importance of influenza vaccination, specifically for single operating units (medical doctors, nurses and medical residents), with a selected audience of subjects sharing the work environment [22, 24].

Furthermore, an improved promotion of web sites and institutional informative sources for influenza and the relative vaccination could be considered as well, for example during the training meetings and through hanging flyers in the operating units [32].

In conclusion, university training on influenza and vaccination programs could be improved by defining multidisciplinary shared educational pathways, and by organizing training courses on the subject during postgraduate courses, with the common aim to improve knowledge and to encourage proactive attitudes of the “future doctors” towards influenza vaccination, certifying it as an evidence-based public heath strategy in preventing hospital-acquired infections. Moreover, future health care providers should be equipped with not just the knowledge, but also the skills in counseling patients regarding the importance of vaccination.
